# The complete chloroplast genome of highbush blueberry (*Vaccinium corymbosum*)

**DOI:** 10.1080/23802359.2021.2009384

**Published:** 2021-12-20

**Authors:** Xiao-Rong Miao, Qiu-Xing Chen, Jun-Qi Niu, Yi-Peng Guo

**Affiliations:** Guangxi Key Laboratory of Agricultural Resources Chemistry and Biotechnology, Yulin Normal University, Yulin, China

**Keywords:** *Vaccinium corymbosum*, chloroplast genome, phylogenetic analysis

## Abstract

Highbush blueberry is a small berry fruit tree belonging to the family Ericaceae and genus *Vaccinium*, which fruit has high nutritional value. High-throughput sequencing technology was applied in this study to sequence and assemble the whole chloroplast genome of the southern highbush blueberry variety sharpblue. The results of the study showed that the circular genome of sharpblue is 170,737 bp in length, and the GC content of the genome was 36.8%. The complete chloroplast genome of sharpblue has consisted of two inverted repeat regions (IRs), a large single-copy region (LSC, 31, 076 bp), and a small single-copy region (SSC, 3, 044 bp). The chloroplast genome contained a total of 144 functional genes, including 100 mRNA genes, eight rRNA genes, and 36 tRNA genes. In addition, *V. corymbosum* and *V. oldhamii* were clustered into one group in this phylogenetic analysis which indicated that they have a close evolutionary relationship. The findings of this investigation are a significant reference source for the phylogeny and evolutionary origin of the Ericaceae family.

Blueberry (*Vaccinium corymbosum* L.) originated in the United States and is the general term for perennial deciduous or evergreen shrubs belonging to the genus *Vaccinium* in the Ericaceae family. Its fruit is rich in a variety of antioxidants, which can slow down aging, and improve the immunity of human. According to plant growth habits, fruit characteristics, and plant height, blueberries are divided into five cultivated groups: northern highbush blueberry, southern highbush blueberry, half-height blueberry, lowbush blueberry, and rabbiteye blueberry. At present, the United States has bred more than 400 blueberry varieties, while China's wild bilberry resources have 91 species, 24 varieties, and two subspecies, but the degree of development and utilization is very low (Xu et al. 2021). To speed up the blueberry breeding process, DNA molecular marker technology is widely used in species relationships, population genetic diversity and genetic map construction (Holá et al. 2017). Through this study, the chloroplast genome of southern highbush blueberry was sequenced and annotated, and the outcome could be used as an important reference for future exploration of species, phylogeny, and evolutionary origin of the Ericaceae family.

The samples of ‘Sharpblue’ southern highbush blueberry (*V. corymbosum* interspecific hybrid) were collected at the Horticulture Training Base of Yulin Normal University, Yulin City, Guangxi Province, China (22°40′41″N, 110°11′39″E). The specimens were stored in the plant herbarium of Yulin Normal University (https://www.ylu.edu.cn/, Jun-Qi Niu, niujunqi3218@163.com) under the voucher number LM202118. The total DNA was extracted from 100 mg fresh young leaves of sharpblue using genomic DNA extraction kit (Tiangen Biotech Co., Ltd., Beijing, China). The quality of extracted DNA was determined with 0.8% agarose gel electrophoresis and quantified by nanodrop 2000c spectrophotometer (Thermo Fisher Scientific, Waltham, MA, USA). The Nextera XT DNA library preparation kit (Illumina Inc, San Diego, CA, USA) was used to construct a cDNA library with an average length of 350 bp and sequenced on the Illumina Novaseq 6000 platform (Novogene Co., Ltd, Beijing, China). The clean data 3.91 GB after quality control filtering was obtained (Patel and Jain 2012), and the average sequencing depth was 529X. According to the chloroplast genome of *V. erythrocarpum* (MW006668.1), *De novo* sequencing data was assembled using SPA software (Bankevich et al. 2012). Nucleotide sequences were annotated using PGA software (Qu et al., 2019). Finally submitted the annotated complete chloroplast genome to the GenBank database, and obtained gene accession number MZ328079.

The complete chloroplast genome of sharpblue is a typical circular double-stranded tetrad structure, having a large single-copy region (LSC), two inverted repeat regions (IRs), and a small single-copy region (SSC), with a length of 31,076 bp, 105,541 bp and 3044 bp, respectively. The total length of the chloroplast genome is 170,737 bp, and has 36.8% GC content. In the current study, a total of 144 genes were annotated including 100 protein-coding genes, 36 tRNA genes, and eight rRNA genes. Among them, 10 genes containing two copies, were *rrn*4.5, *rrn*5, *rrn*16, *rrn*23, tRNA-Ala, tRNA-Ile, tRNA-Arg, tRNA-Asn, ORF302 and *ycf*1, while 15 genes containing one intron, were *atp*F, *ndh*A, *ndh*B, *pet*B, *pet*D, *rpl*16, *rpl*2, *rpo*C1, *rps*16, trnA-UGC, trnI-GAU, trnL-UAA, trnK-UUU, trnG-UCC and trnV-UAC. However, *clp*P and *ycf*3 genes contained two introns and *rps*12 gene had trans-splicing.

For the identification of the evolutionary status of *V. corymbosum*, complete chloroplast genome sequences of 31 Ericales plants were compared using MAFFT 7.48 software (Katoh et al. 2019), and phylogenetic relationship was constructed based on the chloroplast genome sequences by applying neighbor-joining method MEGA7.0 software (Kumar et al. 2016). The phylogenetic tree displayed that *V. corymbosum* and *V. oldhamii* were clustered together, indicating that they have a close evolutionary relationship (Figure 1). The outcome of this study provides a reference for molecular markers, species identification, and phylogenetic studies of the Ericaceae family.

**Figure 1.**
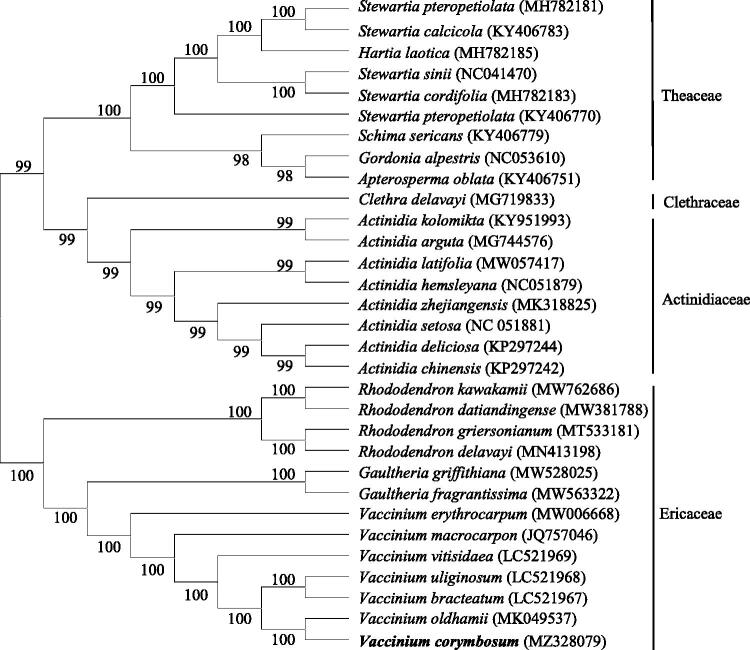
Phylogenetic tree of 31 species based on the neighbor-joining method analysis of the complete chloroplast genome sequences using 1000 bootstrap replicates.

## Data Availability

The chloroplast genome of *V. corymbosum* has been registered in the GenBank database of NCBI (https://www.ncbi.nlm.nih.gov) with the accession number MZ328079. The associated BioProject, SRA, and Bio-Sample numbers are PRJNA732065, SUB9706918, and SAMN19307648, respectively.
